# Optimal Combination of Anodal Transcranial Direct Current Stimulations and Motor Imagery Interventions

**DOI:** 10.1155/2018/5351627

**Published:** 2018-04-02

**Authors:** Elodie Saruco, Franck Di Rienzo, Susana Nunez-Nagy, Miguel A. Rubio-Gonzalez, Ursula Debarnot, Christian Collet, Aymeric Guillot, Arnaud Saimpont

**Affiliations:** ^1^Laboratoire Interuniversitaire de Biologie de la Motricité (EA 7424, LIBM), Univ Lyon, Université Claude Bernard Lyon 1, 69622 Villeurbanne, France; ^2^Universidad de Alcalá, Unidad de Fisioterapia, Campus Universitario, Ctra. Madrid Barcelona, Alcalá de Henares, 28801 Madrid, Spain; ^3^Facultad de Ciencias, Departamento Automática e Informática, UNED, Paseo Senda del Rey 9, 28040 Madrid, Spain; ^4^Institut Universitaire de France, Paris, France

## Abstract

Motor imagery contributes to enhance the (re)learning of motor skills through remapping of cortical networks. Combining motor imagery with anodal transcranial direct-current stimulation (a-tDCS) over the primary motor cortex has further been shown to promote its beneficial effects on postural control. Whether motor imagery should be performed concomitantly to a-tDCS (over depolarized membrane) or consecutively (over changing neurotransmitters activity) remains to be elucidated. In the present study, we measured the performance in a postural control task before and after three experimental conditions. Participants received a-tDCS before (tDCS_Before_), during (tDCS_During_), or both before and during motor imagery training (tDCS_Before + During_). Performance was improved after tDCS_During_, but not after both the tDCS_Before_ and tDCS_Before + During_ conditions. These results support that homeostatic plasticity is likely to operate following a-tDCS through decreasing cortical excitability and that motor imagery should be performed during anodal stimulation for optimum gains.

## 1. Introduction

Motor imagery–the mental simulation of an action–and actual execution of the corresponding movement are known to activate comparable neural networks [1–3]. Based on such partial neural substrates overlap, motor imagery (MI) has been shown to promote the capacity of neurons to adjust their connectivity to the cognitive/behavioral demand, thus eliciting activity-dependent plasticity [4] and significant effects on motor (re)learning [5]. During the last two decades, investigating the beneficial effects of MI on motor function recovery has been the subject of a compelling body of research [6–8]. Of specific interest, MI has been found to facilitate the ability to perform daily activities requiring an adequate postural control in young [9] and elderly persons [10] as well as in patients with stroke [11, 12].

Recent years witnessed a surge of interest in the brain stimulation delivered during MI. Applying anodal transcranial direct-current stimulation (a-tDCS), a non-invasive brain stimulation technique known to increase cortical excitability [13], over the primary motor cortex (M_1_) during MI, has been found to yield additional performance gains in hand motor tasks compared to MI alone [14, 15]. Interestingly, Saruco et al. [16] further reported that combining MI with a-tDCS resulted in greater performance improvements in a task requiring strong postural regulations. Data showed that postural adjustments with low margins for performance improvement, and/or which were particularly difficult to acquire, were enhanced only when MI was combined with a-tDCS.

The effect of a-tDCS on cortical excitability may outlast the stimulation period and persist for up to 90 min [13]. Interestingly, while the modulation of cortical excitability during stimulation stems from resting membrane potential modifications (i.e., depolarization [17]), the after-effects originate from changes in neuromodulators activity (increased intracortical facilitation and decreased intracortical inhibition [18]). Whether a MI session should ideally be scheduled over depolarized neuronal membranes (i.e., during a-tDCS) or during relevant neuromodulatory states (i.e., right after a-tDCS) requires further investigation. So far, research addressing the timing-dependent effects of a-tDCS on motor learning provided inconsistent results. Giacobbe et al. [19] found that motor performance of patients with stroke on a robotic wrist extension task improved when a-tDCS was delivered before training, but stagnated when applied concomitantly to the training session. Likewise, Kuntz et al. [20] observed that training on a digit serial reaction time task right after a-tDCS resulted in performance gains, whereas combining a-tDCS with actual practice had no significant effects on learning. Using a similar paradigm, Kuo et al. [21] reported divergent findings. They found that preconditioning cortical excitability through a-tDCS did not contribute to promote implicit motor learning. Other researchers further demonstrated that applying a-tDCS prior to a sequence-learning task even hindered explicit learning [22], whereas delivering the brain stimulation during actual practice improved performance [23]. To our knowledge, only Sriraman et al. [24] addressed similar timing-dependent effects of a-tDCS on a motor task with lower limbs. They found that delivering a-tDCS during practice better increased motor performance than applying a-tDCS prior to motor practice, without hindering the enhanced retention of motor performance. In addition, whether a further increase of cortical excitability triggered by membrane depolarization (during a-tDCS) on prefacilitated cortical activity (due to a-tDCS after-effects) could provide additional benefits on motor learning also remains unknown.

Aside from the context of physical training, the timing of a-tDCS delivery with reference to MI training has never been considered. The present study was therefore designed to determine whether MI should ideally be scheduled right after, during a-tDCS, or both right after and during a-tDCS.

## 2. Material and Methods

### 2.1. Participants

Sixteen right-handed and right-footed healthy students (7 men and 9 women, mean age 20 ± 2 years) voluntarily participated in this study. Participants had no contraindication to tDCS or a shoe size above 10.5 (US size), allowing a correct use of the postural tool. Each participant gave a written informed consent in agreement with the Declaration of Helsinki [25] before engaging in this double-blind experiment, which was approved by the local research ethics committee of the University.

### 2.2. Experimental Design

MI ability was first assessed using the revised version of the Movement Imagery Questionnaire (MIQ-R [26]). The test addresses the ease to perform MI in the visual and kinesthetic modalities, using four movements (hip abduction, squat jump, arm movement, and forward bending). For each item, participants read a description and physically performed the movement, before imagining through each modality. MI ease was rated on a 7-point Likert-type scale ranging from 1 *(very hard to see/feel)* to 7 *(very easy to see/feel)*. An average score for each modality and one for the entire questionnaire was calculated, with higher score representing greater ease of imaging. Despite its subjective nature, MI vividness assessment through a questionnaire is considered a reliable tool, as suggested in neurophysiological and neuroimaging studies [7]. MI vividness was also controlled after each experimental condition with a 5-point Likert-type scale. Specifically, participants self-reported from 1 (no sensation at all) to 5 (sensation as intense as during the actual execution) the intensity of the sensation perceived (i.e., kinesthetic MI vividness).

**Figure 1:**
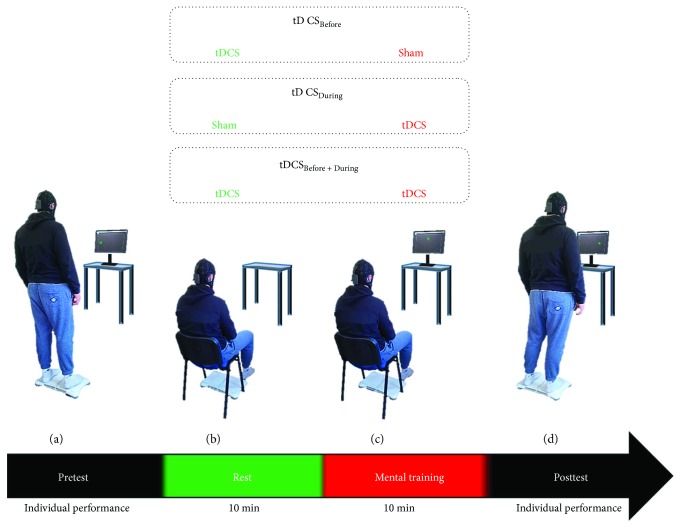
Time course of the experimental design. Participants performed the postural task during the pretest (a), immediately followed by 10 min of rest where participants relaxed while receiving either sham or anodal stimulation over M_1_ (b). A 10 min MI session was then completed while participants received another sham or anodal stimulation (c). Finally, participants performed the posttest (d), which was strictly similar to the pretest.

This double-blinding test-retest involved a MI session during which participants mentally rehearsed a postural task while being subjected to three conditions of M_1_ stimulation. During the pretest, participants were first requested to actually perform a postural task (Figure 1(a)). Right after, they received 10 min of either sham or anodal stimulation and then performed 10 min of MI, which was also combined to a second sham or anodal stimulation. Three experimental conditions were therefore scheduled: (1) a-tDCS, then MI associated with sham stimulation (tDCS_Before_), (2) sham stimulation, then MI associated to a-tDCS (tDCS_During_), and (3) a-tDCS both before and during MI (tDCS_Before + During_) (Figures 1(b) and 1(c)). A posttest session, strictly similar to the pretest, was performed immediately after completing the experimental conditions (Figure 1(d)). All participants randomly performed the three conditions, with a one-week delay between each session to avoid any carry-over effect [27]. Participants benefited from a familiarization with the task before starting the experimentation, allowing them to calibrate their postural skills.

#### 2.2.1. Postural Task

**Figure 2:**
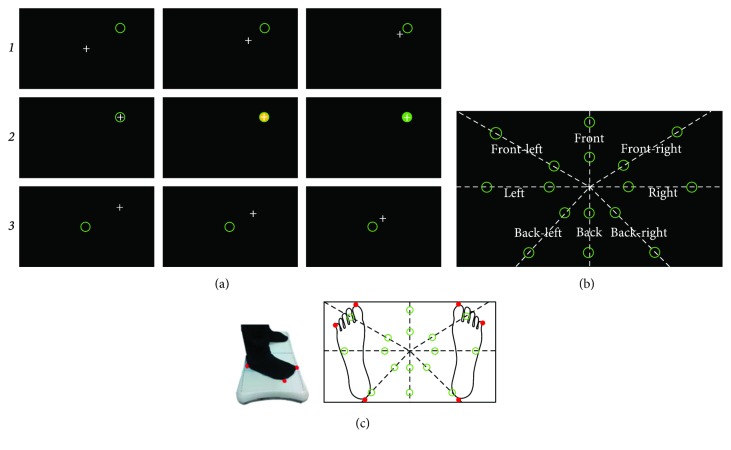
Postural task characteristics. (a_1_) Shift of the CoP (white cross) from a reference position until reaching a randomly assigned target (green circle). (a_2_) The target changed into yellow when the CoP remained within the target during 2 seconds, green after 3 seconds, and then disappeared (i.e., target validated). (a_3_) Participants shift back their CoP to validate a reference target, before the next randomly assigned target appeared. (b) A total of 16 targets appeared on 8 different locations, with two levels of difficulty. Easy and hard targets were, respectively, located at 20% and 50% of the theoretical maximum stability limitation, previously individually delimitated according to the feet positions. (c) Comfortably standing on the Wii Balance Board, coordinates of the heels, and big and pinky toes were used to define the lines on which the targets appeared. Diagonals were calculated with the heel points and half of the distance between big and pinky toe points.

During both the pre- and posttests, participants were required to perform a postural task which consisted in the validation of 16 targets that randomly appeared on a screen [16]. Data were collected from a Wii Balance Board, which reliability as a tool of postural assessment has been validated [28–32]. From a standing position on the balance board, participants received a continuous visual feedback of their center of pressure (CoP) on the screen, represented by a cross. By shifting their CoP without lifting any foot, the cross had to reach and stand into each target area during 3 s to complete its validation (Figure 2(a)). Targets were individually located in 8 different directions with two difficulty levels each, according to the distance from the initial position (Figure 2). Difficulty thresholds were determined according to the sustentation polygon of each participant (Figure 2(c)). After each of the 16 targets (8 easy and 8 hard), a reference target (located in the center) had to be validated before shifting the CoP to the next target (Figure 2(a)). The duration of the task (i.e., validation of the 16 random and 16 reference targets), that depended on participants' individual postural abilities, ranged between 3 and 5 min.

#### 2.2.2. Motor Imagery

As in Saruco et al.'s [16] study, participants performed MI while seating on a chair, positioned exactly at the same place as the Wii Balance Board (i.e., where the pre- and posttests were performed, Figure 1(c)). During 10 consecutive minutes, participants were requested to mentally shift their CoP to the randomly assigned targets that appeared on the screen, which were identical to those presented during both pre- and posttests. Through kinesthetic MI, they were asked to mentally shift and keep their CoP from a standing position into the targets until their validation. Participants remained with their eyes opened during MI. Verbal indication was delivered by the participants at the end of each target validation, so that the experimenter was able to launch the next target. As during the pre- and posttests, a reference target had to be validated between two randomly assigned targets.

#### 2.2.3. Brain Stimulation

Brain stimulation of 1 mA intensity was continuously delivered during 10 min through two saline-soaked sponge electrodes, with a constant-current stimulator (STARSTIM, Neuroelectrics, Barcelona, Spain). Such stimulation charge has been previously shown to induce after-effects on cortical excitability during 1 h [13]. For a bihemispheric stimulation of lower limbs, the 25 cm^2^ anode electrode was fixed at Cz [33], with reference to the international 10/20 system. The 35 cm^2^ cathode electrode was placed at the center of the forehead. This montage was previously shown as being relevant to enhance the beneficial effects of MI on postural control [16]. At the onset of the stimulation, intensity linearly increased during 30 s until reaching 1 mA, then ramped down to 0 during the last 30 s. For a good level of blinding, current also ramped up and down during the first and last 30 s of the sham stimulation, but was nil during MI.

### 2.3. Data Analysis

MIQ-R scores and participants' self-reports of MI vividness were the psychometric variables for MI ability. The time elapsed from the initial contact of the CoP with the target until it was validated (3 seconds inside the target) was the dependent variable for motor performance (Figure 2).

We used R [34], the packages *lme4* [35], and *ARTool* [36] to run a non-parametric analysis of validation times. Due to deviations from normality (visual inspection of Q-Q plots), we implemented a validated aligned rank transformation (ART) procedure [37]. This procedure consists in a preliminary step of data alignment based on the mean estimates of main/interaction effects of a given factorial model, followed by rank assignment. We applied the ART to a mixed linear model with validation times as the response variable. As fixed effects, we included condition (tDCS_Before_, tDCS_During_, and tDCS_Before + During_), test (pretest; posttest), and difficulty (easy and hard targets). As random effect, we entered the by-subject and by-target random intercepts (front, front-right, front-left, back, back-left, and back-right locations). As post hoc investigations, we used contrast tests with ART (least square means difference) and ran a systematic investigation of main and interaction effects. The statistical significance threshold was set up for a type 1 error rate of 5%. We applied Holm's corrections for multiple comparisons to control the false discovery rate [38].

## 3. Results

### 3.1. MI Ability Data

Participants reported good MI ease and vividness with scores just above the median value. MIQ-R global score (M ± SD) was 5.07 ± 0.69. Visual and kinesthetic subscores were 5.52 ± 0.74 and 4.62 ± 0.98, respectively. Mean participants' self-report of MI vividness was 2.96 ± 0.70.

### 3.2. Performance Data

**Figure 3:**
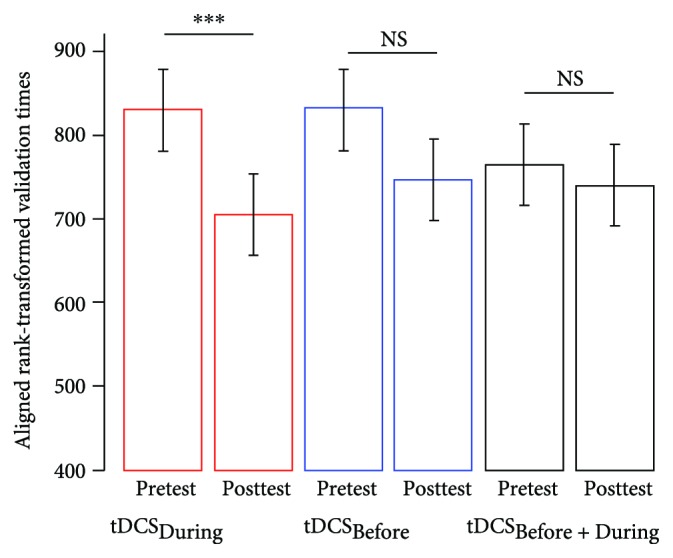
Behavioral outcomes. Least square mean estimates of validation times during the pretest and the posttest across experimental conditions. NS: no statistically significant difference (corrected *p*_(Art)_ values), ^∗∗∗^*p*_(Art)_ value < 0.001.

The linear mixed effect analysis with ART carried on validation times revealed a test × condition interaction (*F*_(2, 1502)_ = 2.60, *p* = 0.03). Validation times were reduced during the posttest (4.25 s ± 1.36) compared to the pretest (4.67 s ± 1.97) in the tDCS_During_ condition (*p*_(ART)_ < 0.001). However, there was no significant difference between pretest and posttest validation times in the tDCS_Before_ (pretest: 4.60 s ± 1.58, posttest: 4.37 s ± 1.71, *p*_(Art)_ = 0.11) and tDCS_Before + During_ (pretest: 4.41 s ± 1.50, posttest: 4.24 s ± 1.09, *p*_(Art)_ = 0.50) conditions (Figure 3). We also observed a test × difficulty interaction (*F*_(2, 1502)_ = 10.92, *p* < 0.001). Difficult targets were validated more rapidly during the posttest than during the pretest (pretest: 5.03 s ± 2.00, posttest: 4.59 s ± 1.67, *p*_(Art)_ < 0.001), while there was no significant difference between the pretest (4.09 s ± 1.13) and the posttest (3.99 s ± 1.00) for easy targets (*p*_(ART)_ = 0.20).

The linear mixed effect analysis with ART also revealed a main effect of test (*F*_(2, 1502)_ = 19.79, *p*_(Art)_ < 0.001) and difficulty (*F*_(2, 1502)_ = 150.80, *p*_(Art)_ < 0.001). Posttest targets were overall validated faster than pretest targets (pretest: 4.56 s ± 1.69, posttest: 4.29 s ± 1.40), and easy targets were validated faster than hard targets (easy: 4.04 s ± 1.07, hard: 4.81 ± 1.86).

## 4. Discussion

This study aimed at investigating the optimal timing of a-tDCS delivery when designing a MI intervention for postural control training. Practically, we tried to determine whether MI should ideally be scheduled right after, during, or both right after and during a-tDCS, in order to evaluate the respective effects of changing neurotransmitters activity and membrane depolarization. While a performance improvement was observed after the concomitant use of a-tDCS and MI (tDCS_During_), applying a-tDCS just before MI (either tDCS_Before_ or tDCS_Before + During_) did not significantly affect the motor performance.

Present data first provided evidence that applying a-tDCS during MI (tDCS_During_) contributed to increase postural regulation to reach a target. This finding confirms previous data supporting that combining MI with a-tDCS can promote motor learning [14–16]. Using a comparable experimental design, Saruco et al. [16] reported that such combination yielded to better performance improvement compared to MI alone, hence supporting that performing MI while membrane potential modulation processes enhance cortical excitability might result in substantial performance gains.

In contrast, performing MI right after a-tDCS (tDCS_Before_) did not significantly improve motor performance, hence suggesting that participants might not benefit from possible synaptic modulations fostering the long-term potentiation [39]. This latter finding is in line with previous results showing no effects [21, 24] or even deleterious effects of a-tDCS on motor learning when stimulation was delivered before physical practice [22, 23]. We thus extend this conclusion to motor learning by MI, confirming similar functional outcomes between actual execution and its mental representation. Interestingly, present result further suggests that applying stimulation just before practice might hinder the benefits observed after a MI session without tDCS [16].

The timing-dependent influence of a-tDCS on motor learning through MI might be explained by homeostatic plasticity processes as proposed by the Bienenstock-Cooper-Munro rule. This stipulates that synaptic modifications, based on the history of their activation, are operated in order to maintain neuronal connectivity within a useful range [40]. Motor learning relies on activity-dependent plasticity which can destabilize neural network properties [41]. The Bienenstock-Cooper-Munro rule postulates that a sliding of the modification threshold (i.e., the level of postsynaptic activity), negatively correlated to the previous neuronal firing rates, operates to avoid such destabilization. In other words, following high levels of neural activity, homeostatic plasticity processes raise the modification threshold for synaptic strengthening, hence fostering inhibition. Regulatory homeostatic plasticity, which occurs in M_1_ following a-tDCS induction [42, 43], might therefore constitute a reliable explanation for the present results. Delivering an excitatory input through MI right after preconditioning a-tDCS might thus have elicited neural inhibition, hence hampering learning. The exact molecular functioning governing such homeostatic plasticity is not yet fully identified. Some studies highlighted the implication of the N-methyl-D-aspartate (excitatory neurotransmitter, NMDA) receptors' activity as an important process involved in homeostatic plasticity [44–46]. Amadi et al. [22] hypothesized that the homeostatic relationship between a-tDCS and motor learning might also occur at the level of gamma-aminobutyric acid- (inhibitory neurotransmitter, GABA-) ergic synapses. This suggests that the sliding of modification threshold may be governed by a modulation of both excitatory and inhibitory nervous processes.

Another original result is the lack of effect of combining a-tDCS with MI after a-tDCS preconditioning (tDCS_Before + During_). As there was a possibility that tDCS_Before_ would positively influence motor learning [20] and as positive effects of tDCS_During_ were anticipated [16], it was relevant to test whether this combination could yield additional improvements. We postulate that further stimulation of the neural state (sparked by tDCS_During_) contributed to trigger the sliding of the modification threshold towards inhibition processes. This result suggests that exogenous modulations of cortical excitability (i.e., a-tDCS) were not sufficient to overcome endogenous homeostatic plasticity processes. Moreover, as we did not find any difference when comparing the level of performance during the posttests of tDCS_Before_ and tDCS_Before + During_ conditions (Figure 1), we hypothesize that a greater input (tDCS_Before + During_) did not lead to a greater reversibility of the neural state (i.e., decrease of the cortical excitability operated by homeostatic processes). Hence, irrespectively of the stimulation charge (tDCS_Before + During_ or tDCS_Before_ only), such processes would operate in a similar manner.

This study has some limitations that should be considered in future experiments. First, we did not include a control of cortical excitability modulations induced by a-tDCS and potential homeostatic plasticity processes. Such interaction has previously been considered by Siebner et al. [42] and Lang et al. [43]. The authors used repetitive transcranial magnetic stimulation (TMS), with the aim to increase cortical excitability. They showed that preconditioning with a-tDCS hampered the increase of cortical excitability induced by repetitive TMS. Measuring the amplitude of motor evoked potential through single-pulse TMS is a reliable method to assess the modulation of cortical excitability. Thus, an interesting perspective would be to collect such data during MI training for further evidence of a-tDCS impact on homeostatic plasticity. Also, assessing the neurobiological factors involved in the relationship between a-tDCS and homeostatic plasticity processes certainly deserves further consideration [22]. From a practical perspective, this study addressed the short-term effects of preconditioning the neural state with a-tDCS, without scheduling a retention test. As Sriraman et al. [24] showed that a-tDCS applied before or during motor learning led to similar performance improvement 24 hours after practice, the effect of time on homeostatic plasticity should be investigated. Finally, there was no control group receiving no stimulation (e.g., sham stimulations before and during MI) in the design, hence preventing from drawing final conclusions regarding the relevance of delivering a-tDCS during MI in order to improve the postural control. However, such direct comparison of the benefits of simultaneous combination of a-tDCS with MI and those observed after MI alone (with sham a-tDCS) were assessed in a previous study [16]. It was found that although MI alone could improve the performance, additional gains were obtained when MI was combined with a-tDCS.

The aim of this experiment was to investigate whether a-tDCS should be applied before, during, or both before and during MI. Data revealed that a-tDCS scheduled before MI (either combined with sham or anodal stimulations) did not significantly improve motor performance, whereas a-tDCS delivered during MI was beneficial. Promising practical applications can be considered in the motor (re)learning domain, as present data support the relevance of applying a-tDCS during MI. This overall suggests that this timing should be regarded for optimal results. Considering the growing interest in brain stimulations and MI, specifically during the neurorehabilitation of patients suffering from locomotor and postural disorders, future studies should investigate in greater details how these two techniques might be adequately combined.
